# A novel technique of a laparoscopic percutaneous guide wire for extraperitoneal internal ring closure in the management of a pediatric communicating hydrocele

**DOI:** 10.3389/fped.2026.1866395

**Published:** 2026-06-05

**Authors:** Guojian Ding, Shuai Sun, Yuming Wang, Yang Xiaofeng, Xinxing Liu, Zhaojiao Cai, Geng Lei, Tingliang Fu, Xiaoliang Xu

**Affiliations:** 1 Department of Pediatric Surgery, Binzhou Medical University Hospital, Binzhou, China; 2 Department of Orthopedics, The Affiliated Dongnan Hospital of Xiamen University, Zhangzhou, China

**Keywords:** children, guide wire, hernia, hydrocele, inguinal, laparoscope, laparoscopy, LPEC

## Abstract

**Objective:**

To investigate the novel technique of a laparoscopic percutaneous guide wire for extraperitoneal closure (LPEC) of a patent processus vaginalis (PPV) of hydrocele in the pediatric age population.

**Methods:**

A retrospective study was conducted on consecutive patients with a pediatric hydrocele between January 2018 and October 2019 to examine our novel laparoscopic percutaneous extraperitoneal closure technique using a guide wire. Data on occult contralateral hydrocele occurrence, operative time, and postoperative complications were collected and analyzed.

**Results:**

A total of 387 patients were included in this study, and their ages ranged from 2.33 to 9.50 years. A total of 65.6% of patients had a unilateral hydrocele and 34.4% had a bilateral hydrocele. The rate of occult contralateral hydrocele was 30.6%. The median operative time for the unilateral hydrocele was 18.6 min and for the bilateral one, it was 27.3 min. The median follow-up period was 24 months, and no complications were reported, except for early postoperative pain in the umbilical port site (*n* = 5) and scrotal edema (*n* = 2).

**Conclusions:**

LPEC using a guide wire was found to be safe and efficacious with less hospital costs for the management of hydrocele in children.

## Introduction

A pediatric hydrocele is a common disorder mainly caused by incomplete obliteration of the processus vaginalis and characterized by fluid collection along the course of the processus (1). The effective surgical intervention is closure of the PPV, which is preferably performed after the patient attains 1 year of age. Because of a spontaneous resolution rate within the first few months of life, surgical intervention for the treatment of this disorder is usually initiated at the age of 1 year (1, 2). The principle for the management of this disorder is to ligate the patent processus vaginalis (PPV) with or without draining the peripheral hydrocele. With the development of minimally invasive surgeries, many laparoscopic techniques, including percutaneous internal ring suturing and single-incision laparoscopic percutaneous extraperitoneal closure (LPEC), have been introduced and have achieved good outcomes because of desirable visual exposure, minimal dissection, fewer intraoperative complications, less recurrence rates, reduced risk of metachronous hydrocele, and improved cosmetic results (3, 4).

**Figure 1:**
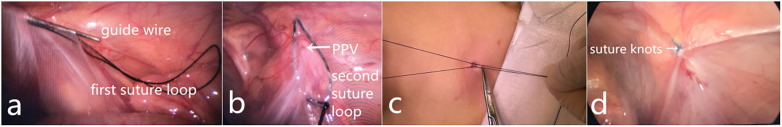
Closure of the PPV at the inguinal ring extraperitoneally. **(a)** The first suture loop was left in the peritoneal cavity under laparoscopic guidance, the Touhy needle was gradually pulled back, and then a home-made guide wire was inserted into the extraperitoneal space through the Tuohy needle; **(b)** After dissecting the lateral retroperitoneum, the second suture loop was passed through the first suture loop; **(c)** Sutures from the second suture loop were pulled out of the abdomen through the medial peritoneum of the PPV; **(d)** The double sutures were tightly tied separately and the knots were just located in the extraperitoneal. PPV, patent processus vaginalis.

In the present study, we describe a technique of guide wire LPEC of the PPV for the treatment of a pediatric hydrocele. The safety and efficacy of this technique for pediatric PPV management are evaluated.

## Materials and methods

The study protocol was approved by the Ethical Research Committee of Binzhou Medical University Hospital, Shandong Province, China. Written consent was obtained from the parents or legal guardians of patients. The medical data of all children who underwent laparoscopic percutaneous extraperitoneal closure of the PPV from January 2018 to October 2019 were retrospectively collected and analyzed.

The inclusion criteria included patients aged 2–10 years with a hydrocele, a female hydrocele, and an intraoperatively confirmed PPV. The exclusion criteria included patients younger than 2 years of age or older than 10 years, patients with inguinal hernia, recurrent inguinal hernia, or undescended testis. The diagnosis was made by examining the medical records of the patients, a physical examination, ultrasonography, or laparoscopic exploration. The clinical data included age, pre- and postoperative diagnosis, length of operation, and intra- and postoperative complications. Postoperative follow-up was implemented at 1 week and 1 and 6 months in the pediatric outpatient clinic. Then, the patients were interviewed over the phone or by WeChat till 24 months.

Because this study was a descriptive case series without a control group, descriptive statistical analysis was primarily used. Continuous variables were expressed as medians (ranges), and categorical variables were presented as frequencies (percentages).

### Surgical instruments

A Tuohy epidural needle, 135 mm in length and 1.6 mm in external diameter, was used as the puncture and suture device. A guide wire was retrofitted from a Kirschner wire, and a stainless-steel Kirschner wire with a diameter of 1.0 mm was cut to a length of 180 mm. Both sharp ends of the Kirschner wire were meticulously ground and polished to create a blunt, atraumatic tip, preventing any potential injury to surrounding tissues. The guide wire can be sterilized by autoclaving and reused.

### Surgical procedure

Under general anesthesia via tracheal intubation or the laryngeal mask airway, the patients were placed in a supine Trendelenburg position. A 5.5 mm trocar for the laparoscope was inserted into the abdominal cavity through a 6-mm transumbilical incision, and the pneumoperitoneum with CO_2_ insufflation was maintained at 8–10 mmHg. Then, a 5 mm laparoscope was entered into the abdominal cavity to inspect the bilateral internal ring. A 2 mm incision was made at the site of the internal ring to introduce a Tuohy epidural needle with an unabsorbable 2–0 silk suture into the extraperitoneal space anterior to the PPV. First, the medial side of the internal ring surrounding the PPV was dissected extraperitoneally, and second, the vas deferens was separated from the retroperitoneum. Hydrodissection with normal saline injection may be helpful to separate this structure from the retroperitoneum, if necessary (5). The suture, along with the Tuohy epidural needle, was left inside the channel and outside the peritoneal cavity under laparoscopic guidance, thereby creating the first intraperitoneal wire loop. The Touhy epidural needle was gradually pulled back to the extraperitoneal space where the needle entered the extraperitoneum. A home-made guide wire tip was inserted into the extraperitoneal space through the Tuohy epidural needle. The needle was pulled out of the incision. Then, the needle with another unabsorbable 2–0 silk suture was inserted into the extraperitoneal space through the guide wire. After the guide wire was removed, the needle with the suture was used to separate the lateral retroperitoneum around the PPV and from the spermatic vessels (Figure 1).

The second suture with the needle passed through the first suture loop and stayed in the abdominal cavity under laparoscopic assistance. The needle was removed, and the first suture loop was designed as a lasso by pulling the extra ends of the first suture; the second suture loop was pulled out of the abdomen through the medial peritoneum of the PPV. The double sutures were tightly tied separately and the knots were just located in the extraperitoneal.

The hydrocele fluid was squeezed into the free peritoneal cavity before the PPV was closed by external manual compression or was percutaneously aspirated with a syringe. If an asymptomatic contralateral PPV was confirmed, the same procedure was performed.

## Results

As shown in Table 1, a total of 387 patients were enrolled in the study, with a median age of 3.75 years (ranged 2.33–9.50 years). Among them, 254 patients (65.6%) had a unilateral hydrocele and 133 (34.4%) had a bilateral hydrocele. The prevalence of the right-sided (61.81%) PPV was significantly higher than that of the left-sided (38.19%) PPV in patients with a unilateral hydrocele. In 366 patients with a unilateral hydrocele detected by a preoperative physical examination or ultrasonography, an occult contralateral PPV was found in 112 patients (30.6%). The hydrocele fluid could be completely flowed into the peritoneal cavity in 87.08% of patients, and percutaneous aspiration was required for the remaining patients (12.92%).

**Table 1:** The results of the LPEC of PPV using a guide wire for a pediatric hydrocele.

Variables	Value
Number of children (*n*)	387
Median ages (years)	3.75 (range, 2.33–9.50)
Side of the hydrocele (*n*)	
Unilateral	254 (65.6%)
Right/left	157/97
Bilateral	133 (34.4%)
Preoperative/intraoperative	21/112
Aspiration of the hydrocele (*n*)	50 (12.92%)
Adding another trocar of 3 mm (*n*)	7 (1.81%)
Postoperative complications (*n*)	7 (1.81%)
Incisional pain at the umbilical port site	5 (1.29%)
Incisional infection	0
Scrotal edema	2 (0.52%)
Recurrence	0
Inflammatory reaction at the suture knot	0
Contralateral metachronous hydrocele	0
Median operative time (min)	
Unilateral	18.6 (range, 14.5–24.2)
Bilateral	27.3 (range, 22.4–35.7)
Median follow-up (months)	24 (range, 12–36)

The LPEC procedures were completed in all patients. The median operative time was 18.6 min (range 14.5–24.2 min) for the unilateral hydrocele and 27.3 min for the bilateral hydrocele (range 22.4–35.7 min). Early postoperative complications included incisional pain in the umbilical port site in five patients (1.29%) and scrotal edema in two patients (0.52%), which resolved within 1 week without any intervention. During a median follow-up of 24 months, no complications or reoccurrence of the hydrocele were observed.

## Discussion

A hydrocele is a common disorder in the pediatric population and its rate of incidence is approximately 5.7% (2). Martin et al. (5) introduced a spermatic cord hydrocele classification of funicular (the peritoneal diverticulum communicating with the peritoneal cavity at the internal inguinal ring) and encysted types (the cyst not communicating with the peritoneal cavity or processus vaginalis) and Chang et al. (6) added a mixed type of spermatic cord hydrocele (the cyst is not communicating with the peritoneal cavity but has a proximally patent processus vaginalis). In the present study, the spermatic cord hydroceles were mainly composed of funicular (85.4%) and mixed types (14.6%), and no encysted type was observed. This finding is consistent with that of Saka's report (7).

The timing of surgical intervention for a pediatric spermatic cord hydrocele is controversial. Spontaneous obliteration of the PPV and self-absorption of the hydrocele may occur in 89% of children with increasing age, and a 2-year observation protocol for patients with hydrocele may be a safe option if no inguinal hernia is present (3, 8). However, some studies have demonstrated that elective operation should be recommended regardless of age (1, 7). In this study, “older than 2 years of age” was considered a surgical indication for a pediatric hydrocele.

Among 366 patients with a unilateral hydrocele diagnosed preoperatively, 112 patients (30.6%) were confirmed to have an occult contralateral PPV under an exploratory laparoscope. This finding is consistent with that of previous reports of rates in the range of 29.03%–63% (1, 9–11). With regard to the management of an occult contralateral PPV, there is no guideline or expert consensus on surgical intervention for this condition (6, 8, 12). However, a contralateral PPV should be considered a risk factor for the development of inguinal hernia beyond the pediatric period, and patients may face the risk of anesthesia exposure. Based on this reasoning, a routine LPEC of an occult contralateral PPV was performed in this study. A prospective randomized study with long-term follow-up is required in the future.

Although this study mainly focused on a pediatric hydrocele, the described guide wire–assisted LPEC technique is equally applicable for, and holds significant value for, the treatment of indirect inguinal hernia in children, as both conditions stem from a patent processus vaginalis. The principles of extraperitoneal dissection and precise ligation are the same.

The LPEC technique for the pediatric inguinal hernia in this study revealed its advantages of less traumatic events, reduced postoperative pain and infection, faster recovery, improved cosmesis, and fewer complications (13). We used a home-made guide wire to make sure that the suture knots were located extraperitoneally to prevent the occurrence of foreign body reaction, infection, granuloma, or knot-related sinus (7) and simplified the procedure by using a single port for camera insertion (3, 12, 14). The risk of injury to the vas deferens and spermatic vessels was minimized with the use of a modified technique of preperitoneal hydrodissection (3, 13). The recurrence rate of LPEC for the inguinal hernia or hydrocele was in the range of 0%–1.4% (1, 15). The learning curve factor, uneven tension of the ligation, integrity of the peritoneum, knot reaction, or ligature loosening are the main causes of recurrence (16). To ensure the integrity of the peritoneum, a 3 mm trocar and clamp that helps separate the peritoneum from the vas deferens is added if necessary. In this study, no complications, including incisional infection, inflammatory reaction at the suture knot, recurrence, and contralateral metachronous hydrocele, were observed, except that seven patients experienced umbilical port site pain or scrotal swelling.

There were some limitations in this study. First, this was a retrospective study conducted at a single institution without a control group. The lack of a control group (e.g., patients undergoing open surgery and the cost for patients using disposable instruments) prevents providing strong evidence through comparisons to illustrate the advantages or disadvantages of this laparoscopic technique. Moreover, the sample size used from a single institution is relatively limited. The choice of the operative procedures depended on the discretion of the surgeon, and therefore, we were not able to rule out selection bias. Further multiple randomized controlled trials are needed in the future.

## Conclusion

Our study demonstrated that the present modified guide wire LPEC is safe and feasible. It can achieve excellent extraperitoneal dissection and precise extraperitoneal ligation of the PPV. The home-made guide wire can relieve the economic burden of patients and the healthcare system.

## Data Availability

The original contributions presented in the study are included in the article/Supplementary Material, further inquiries can be directed to the corresponding authors.
